# Systemic ventricular function in Fontan patients at rest and after exercise at altitude

**DOI:** 10.3389/fped.2022.1084468

**Published:** 2023-01-06

**Authors:** Hannah Quante, Nicole Müller, Julian Alexander Härtel, Thomas Jung, Ursula Manunzio, Johannes Breuer, Ulrike Herberg

**Affiliations:** Department of Pediatric Cardiology, University Hospital Bonn, Bonn, Germany

**Keywords:** Fontan, high altitude, echocardiography, exercise, ventricular funciton

## Abstract

**Objective:**

Physical activity at high altitude is expected to pose risks for patients with Fontan circulation and to impair systemic ventricular function. This study aims to determine the effect of high-altitude hypoxia on ventricular function in Fontan patients at rest and after exercise. We hypothesize that systemic ventricular function deteriorates under hypoxic conditions in Fontan patients.

**Methods:**

In this prospective study, 21 Fontan patients (NYHA class I-II) and 21 age-, gender- and body mass index-matched healthy controls were enrolled (median age 17.9 and 16.9 years). Transthoracic echocardiography was performed at rest, after peak (PE) and after continuous exercise (CE) in normoxia and hypoxia at simulated altitude (2,500 m above sea level). The effect of hypoxia on echocardiographic parameters was quantified by linear mixed-effects models and the difference between normoxia and hypoxia (*Δ*= hypoxia-normoxia).

**Results:**

At rest, cardiac output (CO) estimated by outflow tract velocity time integral × heart rate and annular plane systolic excursion (APSE) were lower in hypoxia compared to normoxia in Fontan patients (CO: *Δ* = −12.0%, n.s.; APSE: *Δ* = −9.6%, *p* < 0.001), an increase was observed in controls (CO: *Δ* = 8.5%, n.s.; APSE: *Δ* = 2.5%, n.s.). Other parameters of systolic and diastolic function did not show relevant changes. After exercise under hypoxic conditions, Fontan patients did not show relevant deterioration of systolic function compared to normoxia. Late, active diastolic filling reflected by A-wave velocity remained unchanged in Fontan patients, but increased in controls. Under hypoxic conditions, CO and workload were higher after CE than PE in Fontan patients (CO: PE *Δ* = 1,530 vs. CE 1630), whereas controls showed higher work load and CO estimates after PE than CE as expected (CO: PE *Δ* = 2,302 vs. CE 2149).

**Conclusion:**

Fontan patients clinically tolerated short-term altitude exposure up to two hours and exercise and showed no consistent deterioration of systolic systemic ventricular function, but parameters of myocardial contractility, heart rate and cardiac output did not increase as observed in controls. This is likely to be multifactorial and may include intrinsic cardiac dysfunction as well as preload inadequacy and the lack of augmented atrial contraction. CE may be better tolerated than PE.

## 1. Introduction

The Fontan operation has been performed for decades as a palliative procedure in children born with a functionally univentricular circulation (1). A growing number of Fontan patients survive into adulthood (1) and wish to participate in ‘normal’ life. This includes excursions and physical activity at high altitude as well as air travel.

Due to the lack of a contractile subpulmonary ventricle, the Fontan circulation is dependent on low pulmonary vascular resistance to preserve ventricular preload (1), and adequate ventricular function of the systemic ventricle (2). At heights of 2,500 m above sea level (asl), the partial pressure of inspired oxygen drops, corresponding to a decline in fraction of inspired oxygen from ∼21% to ∼15% (3). This hypoxia may induce pulmonary vasoconstriction (4). Consequently, pulmonary vascular resistance may rise, which can lead to a significant decline in cardiac output in Fontan patients (1, 5). This is why high-altitude exposure is expected to pose a risk to patients with Fontan circulation. Moreover, during exercise, cardiac output needs to be augmented (6) putting even higher stresses on the Fontan circulation.

Previous studies reported that short-term exposure and exercise at high altitude was tolerated by Fontan patients (7, 8). These studies investigated the impact of short-term altitude exposure and exercise on hemodynamic and pulmonary responses in Fontan patients.

This study aims to determine to what extent systemic ventricular function changes at rest and after various forms of exercise at simulated altitude in Fontan patients compared to healthy controls. We hypothesize that systemic ventricular function in Fontan patients deteriorates under hypoxic conditions compared to normoxia and compared to healthy controls.

## 2. Materials and methods

### 2.1. Study population

We conducted a prospective, single-center, observational study. Patients with Fontan circulation in good current health status (New York Heart Association (NYHA) class I or II) were recruited. Exclusion criteria were failing Fontan, inability to perform bicycle ergometry, pregnancy, and age below 14 years. Additionally, age-, gender- and body mass index (BMI)-matched healthy controls were enrolled, but those with cardiovascular disorders, athlete status or smoking history were excluded.

### 2.2. Study design and protocol

**Figure 1:**
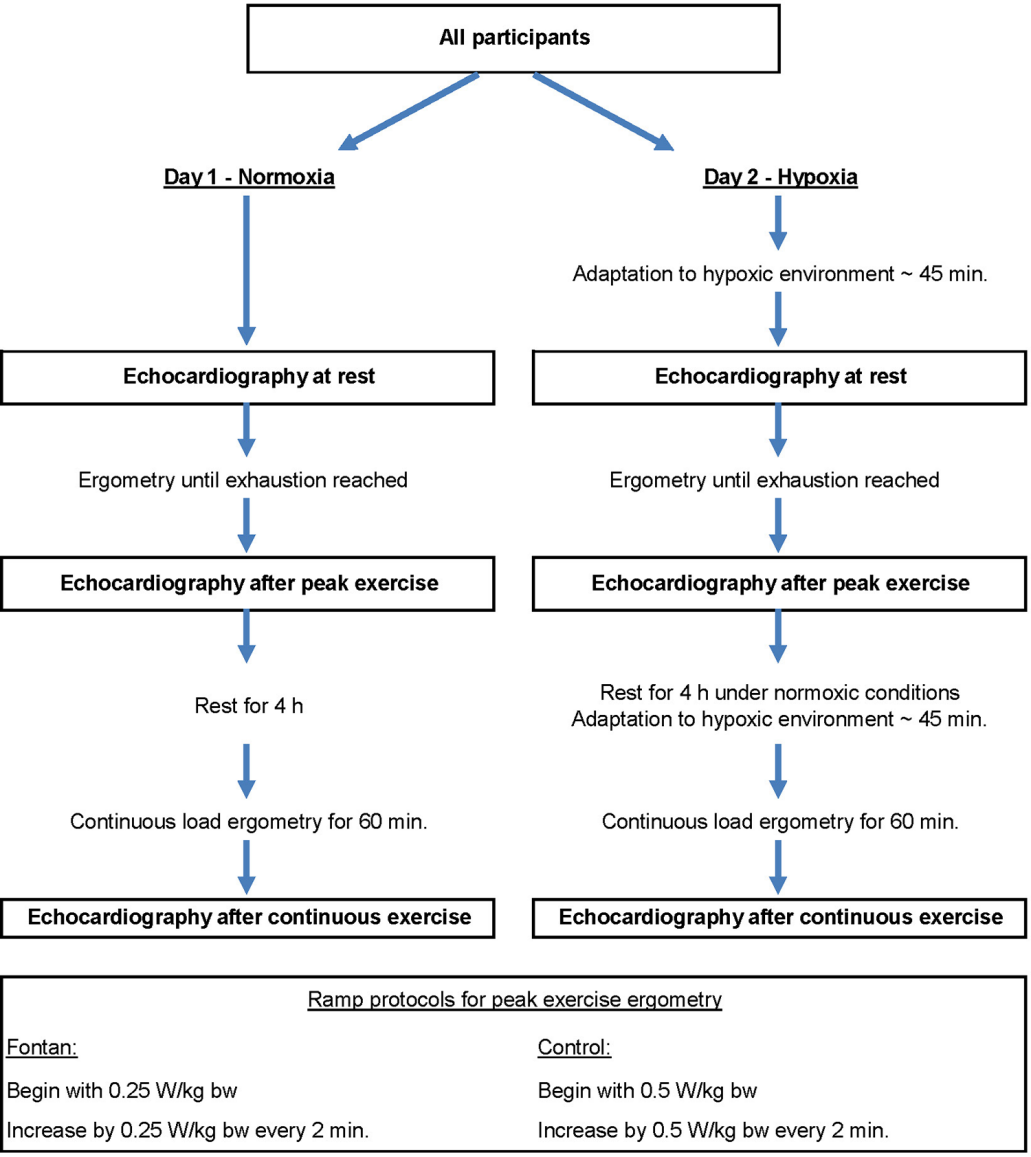
Study protocol. W = Watt; kg bw = kilogram body weight.

All participants underwent physical examination including weight, height, blood pressure, oxygen saturation and resting heart rate. Medical records were reviewed for ventricular anatomy and surgical history. Transthoracic echocardiographic imaging was performed at rest, after peak exercise (PE) and after continuous exercise (CE) under conditions of normoxia at ambient air breathing, and hypoxia at simulated altitude in all participants (Figure 1). Imaging was conducted in supine position after exercise as preliminary echocardiographic examinations during ergometry did not yield evaluable results in Fontan patients. Measurements in normoxia and hypoxia were obtained within a mean time period of 14 days.

To simulate an altitude of 2,500 m asl, a normobaric altitude chamber (Höhenbalance, Going, Austria) was used. Ambient oxygen was reduced to 15.2% and nitrogen increased to 84.8% at constant air pressure (∼ 1,013 hPa).

Exercise testing was conducted using a sitting bicycle ergometer (ERG911 Plus, Ergosana GmbH Schiller, Bitz, Germany). Different ramp protocols were applied in the Fontan and control group to achieve similar subjective loading in a comparable time period (Figure 1) (9). Peak exercise was stopped when subjective exhaustion (BORG-scale >16) was reached or a pedal frequency of 60 revolutions per minute could not be sustained. Continuous exercise, performed for 60 min, was conducted below the anaerobic threshold.

The institutional ethical review board approved the study (application number 335/14) and written informed consent was obtained from all participants and/or their guardian. The study protocol conformed to the principles of the Declaration of Helsinki.

### 2.3. Echocardiographic image acquisition and analysis

All transthoracic echocardiographic studies were performed by experienced senior pediatric cardiologists following a standardized protocol. Images were conducted using a Philips iE33 ultrasound machine with X5–1 transducer (Philips Healthcare, Andover, MA, United States), which was appropriate for the age and weight of all participants.

Analysis was performed off-line using TomTec version 4.7 (TomTec, Unterschleissheim, Germany). Images with inadequate quality were excluded. All measurements were conducted in at least two different cardiac cycles and the results were averaged. In both groups, only the systemic ventricle was used for analysis. In healthy controls, the left ventricle was assessed, whereas the dominant ventricle was measured in the Fontan group.

Analyzing ventricular function by echocardiography is challenging in Fontan patients due to the complex geometry and morphology of the ventricles, therefore echocardiographic parameters suitable for the assessment of both uni- and biventricular circulations were selected and are displayed in Supplementary Table S1.

Ventricular area measurements were obtained from the apical four-chamber view (A4CV) by the Area-Length method (10). Length change was calculated as (end-diastolic length–end-systolic length)/end-diastolic length×100% and fractional area change (FAC) as (end-diastolic area–end-systolic area)/end-diastolic area×100% (10). The velocity time integral (VTI) was measured by pulsed wave Doppler (PW) over the aortic or neo-aortic valve (11). An electrocardiogram connected to the ultrasound machine recorded heart rate (HR) during imaging. The product of VTI and HR at the time of VTI measurement was regarded as a surrogate of cardiac output (CO) (12). Annular plane systolic excursion (APSE) was obtained by M-mode at the lateral tricuspid or mitral annulus as appropriate (13, 14). E- and A-waves were measured by PW at the dominant atrioventricular valve (15). If E- and A-wave fusion occurred, only the E-wave was assessed (16). Tissue Doppler imaging (TDI) (e', a', s') was performed at the free wall of the systemic ventricle (17) corresponding to the lateral wall in healthy controls. In double inlet left ventricles, the lateral wall was chosen. Values of E-wave were divided by e' to compute E/e' ratio. Time intervals measured by TDI include isovolumic contraction time (IVCT), isovolumic relaxation time (IVRT) and ejection time (ET). Myocardial performance index (MPI) was calculated as (IVCT + IVRT)/ET (18). Further, IVRT was divided by RR interval to correct for heart rate (19). Inferior vena cava collapsibility index (IVC CI) was calculated as (maximal diameter–minimal diameter)/maximal diameter (20). The mentioned diameters were obtained by M-Mode (20). Global longitudinal strain (GLS) was measured by two-dimensional speckle tracking from the A4CV using 2D cardiac performance analysis (TomTec version 4.7, Unterschleissheim, Germany). The endocardial border of the systemic ventricle was semi-automatically traced and manually corrected if necessary.

We focused on the following parameters: APSE, s', GLS, HR, VTI, CO, E-wave velocity, A-wave velocity, E/e'.

### 2.4. Statistical analysis

Statistical analysis was conducted using SPSS version 25.0 for Windows (IBM SPSS Statistics, United States). Data are expressed as mean ± standard deviation, median (interquartile range) or counts (percentages) as appropriate. Testing for normality was performed using Q-Q plots. For baseline characteristic comparisons between groups, paired t-tests or Wilcoxon-tests were used as appropriate. In each group, we analyzed the effects of oxygen level and exercise level by linear mixed-effects models (LMM) for all echocardiographic parameters. To evaluate whether the effect of exercise level differs between the two oxygen levels, the interaction term of exercise level and oxygen level was included in all regression models. We accounted for repeated measurements within the study participants by subject-specific intercepts (as random effects). We corrected for multiple testing by Bonferroni correction. A *p*-value of 0.002 was considered statistically significant. To quantify the effect of high-altitude hypoxia on each parameter, mean predicted difference between normoxia and hypoxia was calculated for all exercise levels in each group (*Δ*= hypoxia–normoxia) (*Δ*_r _= at rest, *Δ*_p _= after peak exercise, *Δ*_c _= after continuous exercise). Furthermore, the relative difference was calculated as *Δ*(%) = ((hypoxia–normoxia)/normoxia) × 100%.

*Intra- and interobserver variability*

Ten echocardiographic studies of each group were randomly selected for analysis. For intraobserver variability assessment, single measurements were repeated by the same investigator after 6 weeks. Measurements were repeated by a second observer blinded to the previous results to determine interobserver variability. Intra- and interobserver variability was expressed by intra-class correlation.

## 3. Results

### 3.1. Study population

21 Fontan patients and 21 healthy controls were enrolled. The participants' characteristics are shown in Table 1. All participants were able to perform continuous and peak exercise testing until exhaustion both at sea level and at simulated high altitude with no additional symptoms declared. There were two subjects who did not participate in either normoxic or hypoxic examinations due to time constraints. Oxygen saturation under normoxic conditions was reduced in the Fontan group compared to controls (95.0% (93.0–96.8) vs. 99.0% (98.5–100.0), *p* < 0.001). 2 Fontan patients with fenestration were included in this study showing oxygen saturations of 90% and 85% at rest in normoxia and 88% and 77% at rest in hypoxia. These values were within the range of oxygen saturation in the rest of the Fontan group without fenestration. Fontan patients achieved a mean peak load of 112.7 ± 31.9 Watts (W) in normoxia compared to 209.0 ± 52.3 W in healthy controls. In hypoxia, mean peak loadings of 104.2 ± 29.8 W (Fontan) and 194.9 ± 45.7 W (controls) were achieved. On continuous exercise in normoxia, mean loadings of 42.5 ± 10.4 W were measured in Fontan patients compared to 79.9 ± 22.4 W in healthy controls. Under hypoxic conditions, mean loadings of 40.6 ± 12.8 W (Fontan) and 62.7 ± 18.7 W (controls) were achieved.

**Table 1:** Demographic data.

	Fontan (*n* = 21)	Control (*n* = 21)	*p*-value Fontan vs. Control
Age at study (years)	17.9 (16.1-23.9)	16.9 (15.9-25.2)	0.263
Female	9 (42.9%)	9 (42.9%)	
Height (cm)	166.3 ± 9.1	172.0 ± 10.0	0.001
Weight (kg)	63.9 ± 14.3	67.2 ± 14.0	0.114
BMI (kg/m²)	22.9 ± 3.8	22.5 ± 3.2	0.356
Body surface area (Haycock)	1.72 ± 0.23	1.79 ± 0.23	0.035
Resting heart rate (bpm)	63.7 ± 12.3	64.4 ± 9.1	0.831
Saturation in normoxia (%)	95.0 (93.0-96.8)	99.0 (98.5-100.0)	<0.001
Diastolic blood pressure (mmHg)	63.5 ± 10.3	65.4 ± 7.1	0.501
Systolic blood pressure (mmHg)	114.2 ± 9.5	116.8 ± 7.8	0.356
Mean arterial pressure (mmHg)	80.4 ± 9.5	82.5 ± 6.2	0.424
Pacemaker	3 (14.3%)		
*Systemic ventricle*			
LV	8 (38.1%)	21 (100%)	
RV	13 (61.9%)		
Rudimentary ventricle	4		
Open fenestration	2		
*Cardiac defects*			
Hypoplastic left heart syndrome	6 (28.6%)		
Hypoplastic left heart complex	1 (4.8%)		
Hypoplastic left ventricle with TGA	6 (28.6%)		
Tricuspid atresia	3 (14.3%)		
Double inlet left ventricle	5 (23.8%)		
*Type of Fontan connection*			
TCPC lateral tunnel	16 (76.2%)		
TCPC extracardiac conduit	5 (23.8%)		
*ECG rhythms at rest in normoxia*			
Sinus rhythm	14 (66.7%)		
Pacemaker rhythm	3 (14.3%)		
Atrioventricular junctional rhythm	2 (9.5%)		
Atrial rhythm	1 (4.8%)		
*Medication*			
Cardioselective *β*-blocker	6 (28.6%)		
ACE inhibitors	10 (47.6%)		
Age at Fontan operation (years)	2.9 (2.1-4.4)		

Data are expressed as mean ± SD, median (IQR) or numbers (percentages). The group ‘hypoplastic left ventricle with TGA’ summarizes the following cardiac defects: 1 × Double outlet right ventricle and pulmonary atresia, 1 × heterotaxia-syndrome, 3 × pulmonary atresia. The systemic ventricle is the RV but with TGA. A Norwood operation was not needed. BMI, body mass index; bpm, beats per minute; LV, left ventricle; RV, right ventricle; TGA, transposition of the great arteries; TCPC, total cavo-pulmonary connection; ACE, angiotensin-converting enzyme.

### 3.2. Echocardiographic measurements

Results of echocardiographic parameters of the Fontan and control group are displayed in Supplementary Table S2. As expected, most of the systolic and diastolic parameters were reduced in Fontan patients compared to controls: FAC, VTI, CO, APSE, s', GLS, E, e', IVC CI.

The aim of our study was to determine the changes of cardiac performance in hypoxia at rest and after peak as well as continuous exercise compared to normoxia. Hence, we focused on the statistical comparison of the effects of exercise level and oxygen level on echocardiographic parameters within the Fontan and the control group. Results of selected LMM analyses are summarized in Table 2.

**Table 2:** Effects of exercise level and oxygen level on selected echocardiographic parameters in Fontan patients and healthy controls.

		At rest			After peak exercise			After continuous exercise					
		Normoxia	Hypoxia	*Δ*_r_ (%)	Normoxia	Hypoxia	*Δ*_p_ (%)	Normoxia	Hypoxia	*Δ*_c_ (%)	*p*-value^a^	*p*-value^b^	*p*-value^c^
APSE (mm)	Fontan	12.80 ± 0.89	11.57 ± 0.88	−9.6%	13.21 ± 0.89	10.78 ± 0.88	−18.4%	13.70 ± 0.90	11.70 ± 0.89	−14.6%	<0.001*	0.157	0.805
	Control	16.67 ± 0.97	17.09 ± 0.96	2.5%	16.09 ± 0.97	15.07 ± 0.98	−6.3%	16.32 ± 0.96	17.63 ± 0.95	8.0%	0.765	0.006	0.116
s’ (cm/s)	Fontan	6.67 ± 0.57	6.84 ± 0.55	2.5%	7.42 ± 0.57	7.69 ± 0.56	3.6%	8.02 ± 0.60	7.60 ± 0.57	−5.2%	0.645	0.011	0.831
	Control	10.38 ± 0.79	11.14 ± 0.79	7.3%	12.72 ± 0.79	13.2 ± 0.79	3.8%	11.98 ± 0.80	12.50 ± 0.79	4.3%	0.714	<0.001*	0.953
GLS (%)	Fontan	−22.81 ± 1.36	−20.49 ± 1.34	10.2%	−22.12 ± 1.36	−20.91 ± 1.34	5.5%	−20.71 ± 1.38	−20.77 ± 1.34	−0.3%	0.690	0.380	0.273
	Control	−22.61 ± 1.08	−22.17 ± 1.08	1.9%	−23.53 ± 1.07	−21.94 ± 1.11	6.8%	−22.91 ± 1.08	−23.03 ± 1.08	−0.5%	0.149	0.602	0.310
VTI (cm)	Fontan	21.85 ± 1.38	20.25 ± 1.43	−7.3%	21.15 ± 1.40	19.41 ± 1.39	−8.2%	18.83 ± 1.43	19.68 ± 1.40	4.5%	0.280	0.580	0.692
	Control	23.18 ± 1.35	24.20 ± 1.27	4.4%	21.26 ± 1.39	23.47 ± 1.26	10.4%	24.39 ± 1.27	25.17 ± 1.27	3.2%	0.095	0.018	0.685
CO (VTIxHR)	Fontan	1472 ± 113	1296 ± 118	−12.0%	1622 ± 115	1530 ± 114	−5.6%	1479 ± 118	1630 ± 115	10.2%	0.482	<0.001*	0.284
	Control	1590 ± 144	1725 ± 136	8.5%	2031 ± 149	2302 ± 135	13.4%	2016 ± 136	2149 ± 136	6.6%	0.023	<0.001*	0.636
Area systole (cm^2^)	Fontan	19.53 ± 0.83	20.71 ± 0.84	6.0%	19.56 ± 0.83	20.08 ± 0.84	2.7%	19.20 ± 0.85	18.78 ± 0.84	−2.2%	0.108	0.067	0.036
	Control	19.80 ± 0.79	19.41 ± 0.8	−2.0%	18.18 ± 0.79	19.56 ± 0.8	7.6%	19.03 ± 0.79	18.49 ± 0.8	−2.8%	0.892	0.040	0.059
Area diastole (cm^2^)	Fontan	33.07 ± 1.21	34.17 ± 1.22	3.3%	32.98 ± 1.21	33.59 ± 1.22	1.9%	33.02 ± 1.23	32.87 ± 1.22	−0.5%	0.507	0.786	0.307
	Control	33.51 ± 0.98	32.82 ± 0.99	−2.1%	31.75 ± 0.97	33.18 ± 0.99	4.5%	32.68 ± 0.97	32.28 ± 0.99	−1.2%	0.890	0.146	0.139
A-wave (m/s)	Fontan	0.48 ± 0.06	0.48 ± 0.06	0.0%	0.54 ± 0.06	0.53 ± 0.06	−1.9%	0.52 ± 0.06	0.60 ± 0.06	15.4%	0.426	0.049	0.393
	Control	0.45 ± 0.06	0.51 ± 0.06	13.3%	0.72 ± 0.06	0.82 ± 0.06	13.9%	0.63 ± 0.06	0.57 ± 0.06	−9.5%	0.248	<0.001*	0.047
E-wave (m/s)	Fontan	0.74 ± 0.05	0.76 ± 0.05	2.7%	0.83 ± 0.05	0.81 ± 0.05	−2.4%	0.80 ± 0.05	0.79 ± 0.05	−1.3%	0.630	0.004	0.721
	Control	0.94 ± 0.05	0.93 ± 0.05	−1.1%	0.90 ± 0.05	0.89 ± 0.05	−1.1%	0.90 ± 0.05	0.92 ± 0.05	2.2%	0.757	0.070	0.681
E/e'	Fontan	7.28 ± 0.78	7.54 ± 0.77	3.6%	7.35 ± 0.80	6.35 ± 0.78	−13.6%	7.04 ± 0.81	6.37 ± 0.81	−9.5%	0.732	0.356	0.498
	Control	5.45 ± 0.35	5.57 ± 0.35	2.2%	5.42 ± 0.35	5.31 ± 0.35	−2.0%	5.30 ± 0.35	5.53 ± 0.35	4.3%	0.293	0.490	0.784

Data are presented as mean predicted values ± standard error or percentage as appropriate.

*Δ*(%) = ((hypoxia–normoxia)/normoxia) × 100%.

^a^ *p*-value: main effect hypoxia.

^b^ *p*-value: main effect exercise level.

^c^ *p*-value: interaction effect (hypoxia*exercise level).

* *p*-values <0.002 are considered statistically significant.

#### 3.2.1. Effect of high-altitude hypoxia on systemic ventricular function at rest

**Figure 2:**
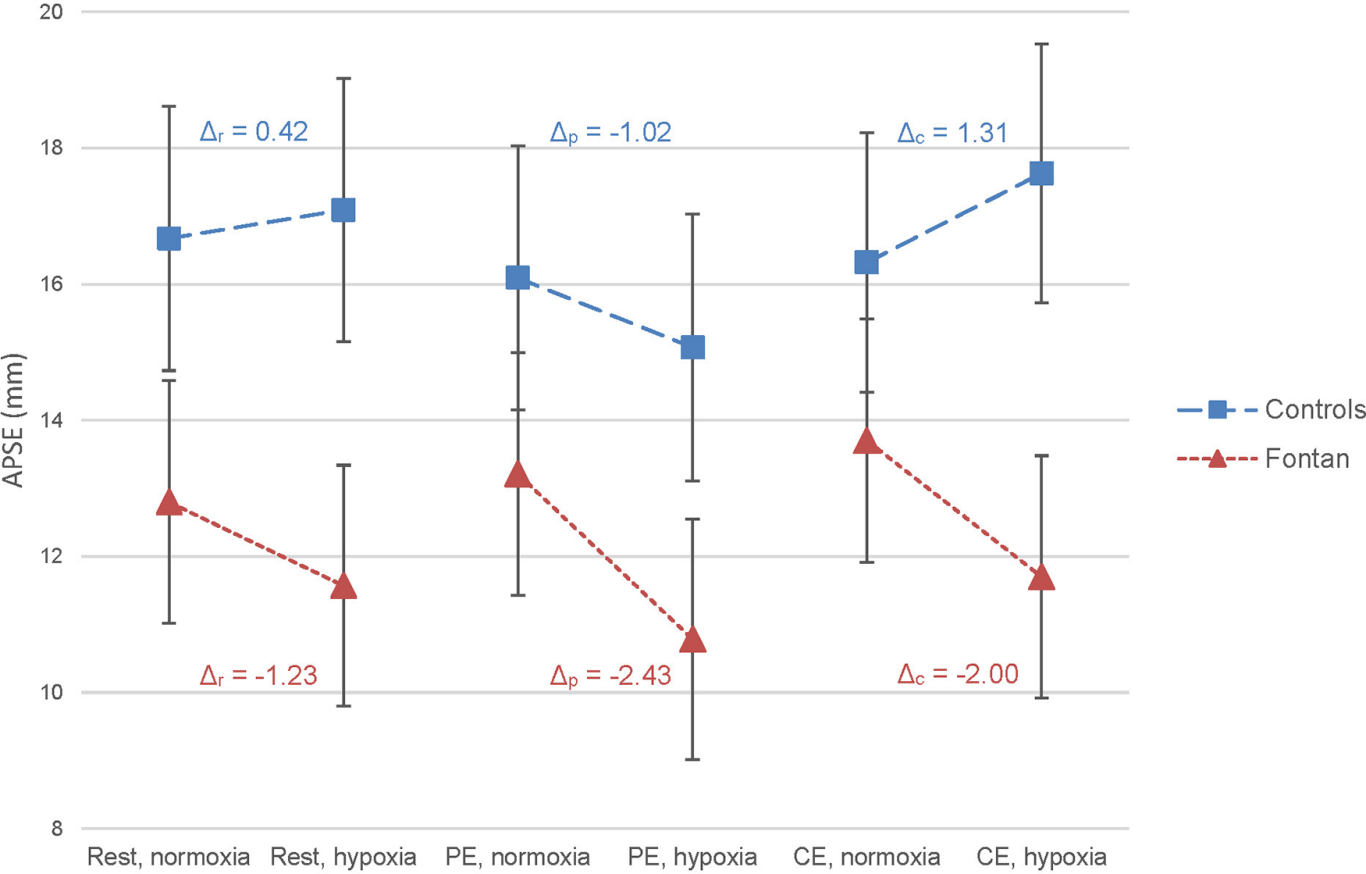
Effect of exercise and hypoxia on annular plane systolic excursion (APSE) in Fontan patients and controls analyzed by linear mixed-effects model (LMM). The figure displays the mean predicted values with 95% confidence interval in the Fontan group (red triangle) and in the control group (blue square) as calculated by LMM. On the x-axis, the studied conditions are presented. Within an exercise level and group, the changes in mean predicted value between normoxia and hypoxia were visually linked by dashed lines and numerically corroborated by the calculation of *Δ* (*Δ* = (mean predicted APSE in hypoxia – mean predicted APSE in normoxia) in mm). *Δ*_r_ = at rest, *Δ*_p_ = after peak exercise, *Δ*_c_ = after continuous exercise. In Fontan patients, hypoxia showed a significant effect on APSE (*p* < 0.001).

Except for s', echocardiographic parameters of systolic function decreased at rest in hypoxia compared to normoxia in the Fontan group. The observed changes of s' (Supplementary Figure S1), FAC, CO and GLS (Supplementary Figure S2) were mild and without clinical relevance. However, a statistically significant negative effect of hypoxia was found on APSE (*Δ*_r _= −9.6%, *p* < 0.001) (Figure 2). In contrast to the Fontan group, parameters of systolic function moderately increased in the control group (APSE, s', VTI, CO) although this was not statistically significant. Parameters of diastolic function did not considerably change between normoxia and hypoxia in both groups.

#### 3.2.2. Effect of high-altitude hypoxia on systemic ventricular function after peak and continuous exercise

**Figure 3:**
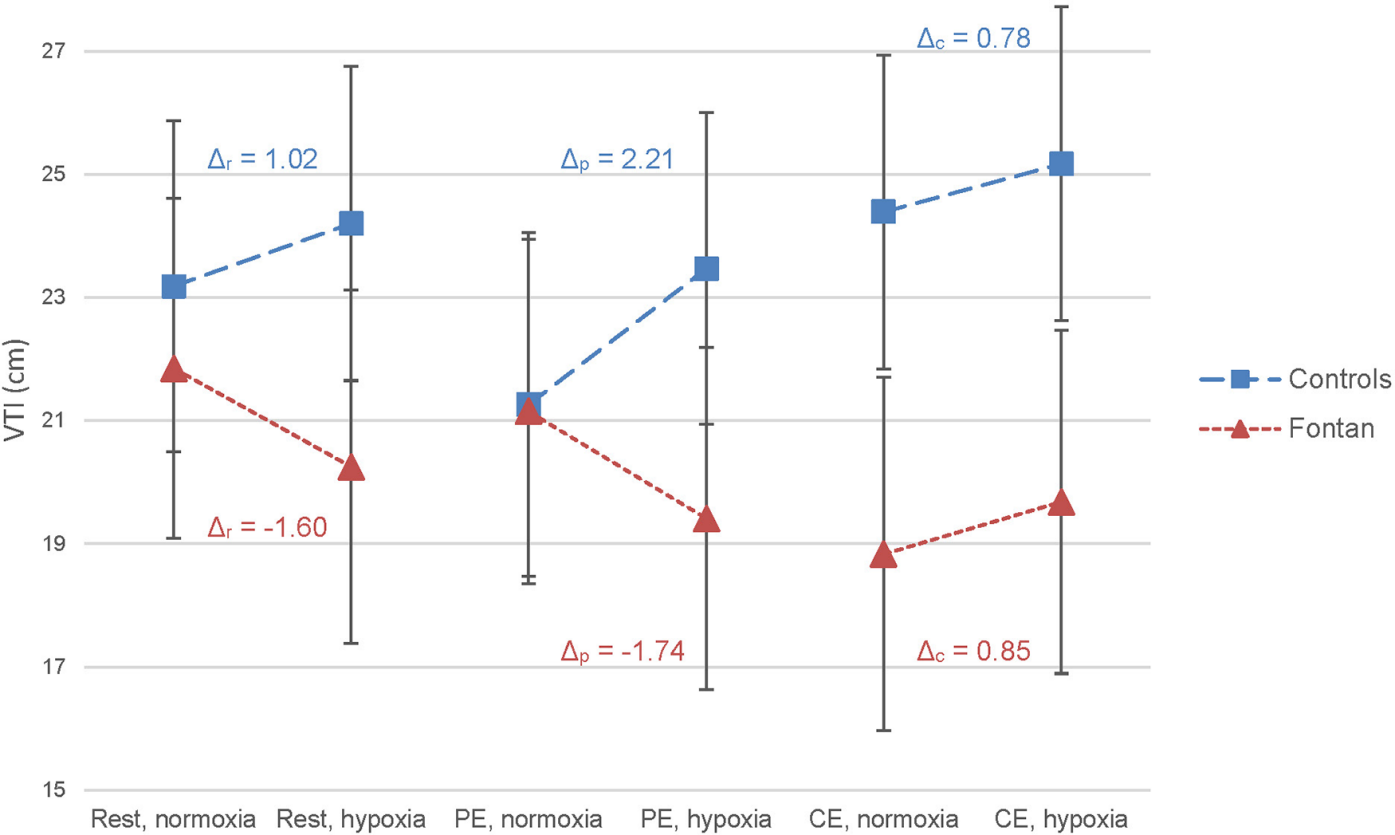
Effect of exercise and hypoxia on velocity time integral (VTI) in Fontan patients and controls analyzed by LMM. No statistically significant effects of exercise or hypoxia on VTI were found in either group. *Δ* = (mean predicted VTI in hypoxia – mean predicted VTI in normoxia) in cm; calculated for each exercise level and group.

**Figure 4:**
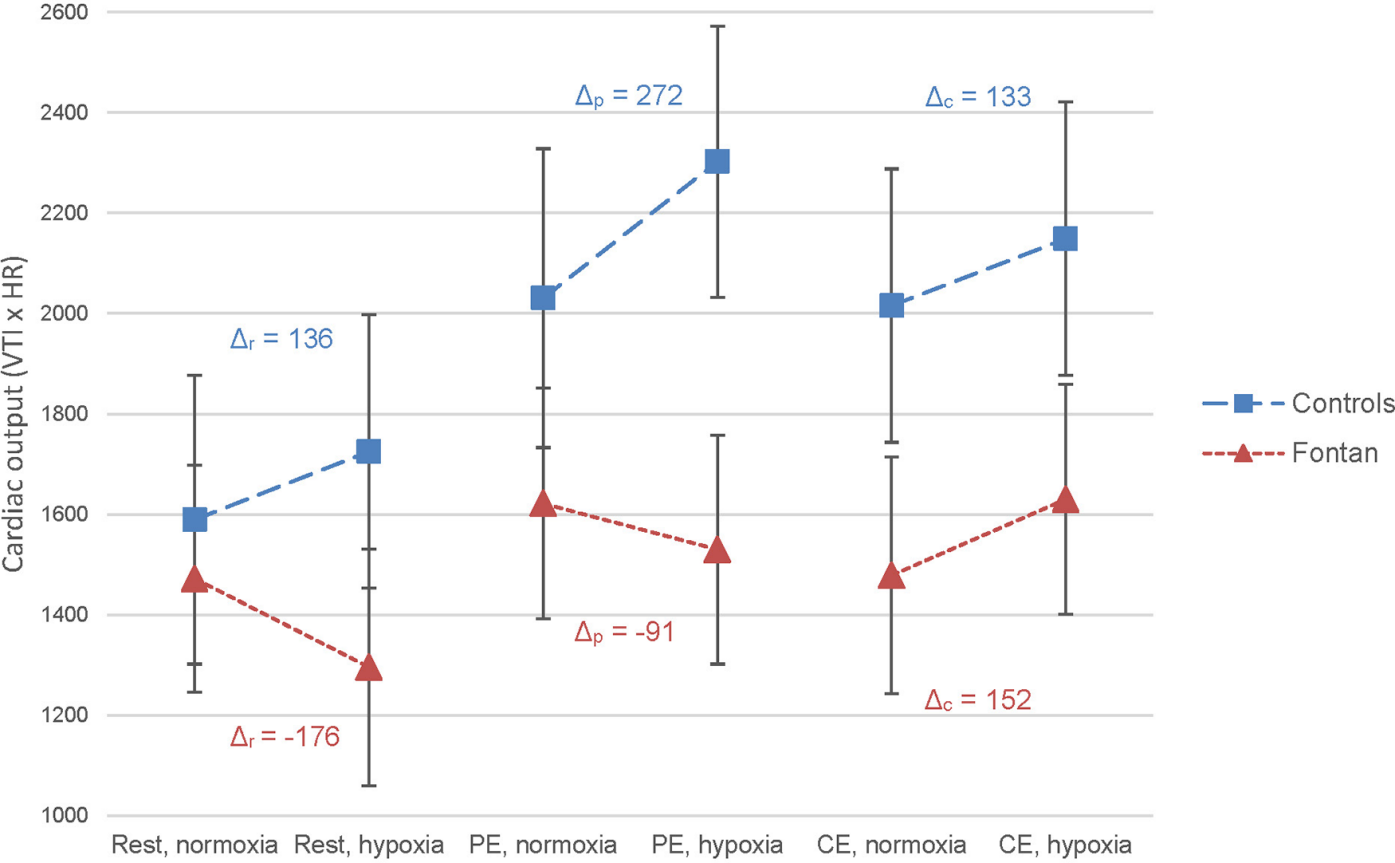
Effect of exercise and hypoxia on cardiac output (CO) estimated by velocity time integral × heart rate in Fontan patients and controls analyzed by LMM. A statistically significant effect of exercise on CO was found in Fontan patients and healthy controls (*p* < 0.001). *Δ* = (mean predicted CO in hypoxia – mean predicted CO in normoxia); calculated for each exercise level and group.

In the control group, a moderate rise of systolic function was observed after peak exercise in hypoxia compared to normoxia (s', VTI (Figure 3), CO). In the Fontan group, surrogate markers for systolic function did not show relevant deterioration after exercise under hypoxic conditions compared to normoxia. Remarkably, CO in Fontan patients was higher after CE than PE in hypoxia (*Δ* = 1,630 vs. 1,530), whereas healthy controls showed higher CO estimates after PE as expected (*Δ* = 2,302 vs. 2,149) (Figure 4). Parameters of diastolic function did not considerably change between normoxia and hypoxia after PE in both groups except for an increase in A-wave in the control group (*Δ*_p _= 13.9%, *p* = 0.248), which was not statistically significant (Supplementary Figures S3–S5).

### 3.3. Feasibility and reproducibility

Due to imaging artefacts immediately after exercise, 80.5% of the parameters could be analyzed on average (ranging from 65.3% to 87.8%).

Results of intra- and interobserver variability analysis are displayed in Supplementary Table S3. Both intraobserver and interobserver variability were within reasonable range.

## 4. Discussion

Summary of the most important findings:

• Short-term exposure to high-altitude hypoxia and exercise was clinically tolerated by Fontan patients.
• No consistent decline in echocardiographic parameters was observed in the Fontan group at simulated altitude.
• Increased values of myocardial contractility, heart rate and cardiac output at rest and after peak exercise were not observed in Fontan patients as in healthy controls.

This study aimed to evaluate the effect of high-altitude hypoxia (2,500 m asl) on cardiac function in Fontan patients at rest and after peak as well as continuous exercise using echocardiography. Contrary to our hypothesis assuming that systemic ventricular function deteriorates under hypoxic conditions in Fontan patients, a consistent decline in echocardiographic parameters was not seen in hypoxia in this group. In fact, variable changes of functional parameters were observed. In comparison to the control group, parameters of myocardial contractility, heart rate and cardiac output did not increase as expected under hypoxic conditions in Fontan patients. However, short-term exposure to these circumstances was clinically tolerated.

Several studies evaluating systemic ventricular function at rest and during exercise by echocardiography in normoxia exist in Fontan patients (21, 22). Yamazaki et al. (21), Tomkiewicz et al. (22) and our study showed that echocardiographic parameters were reduced in patients with single ventricle circulation compared to biventricular circulation.

Hence, the functionally univentricular circulation has been hypothesized to have a poorer adaptation to the changing requirements of high-altitude hypoxia as well as exercise. This stems from the following physiological considerations: Partial pressure of inspired oxygen declines progressively with increasing altitude inducing pulmonary vasoconstriction (3, 4). As Fontan patients rely on passive pulmonary perfusion, factors increasing pulmonary vascular resistance such as hypoxic pulmonary vasoconstriction are expected to lead to a decline in preload, ventricular function and cardiac output (1, 5). However, Staempfli et al. examined the exercise capacity and pulmonary blood flow at low and high altitude and observed a 2.0-fold increase in pulmonary blood flow in Fontan patients and a 2.5-fold increase in the control group during submaximal exercise at high altitude (7). Likewise, Takken et al. found a comparable impact of high altitude on hemodynamic and pulmonary responses in Fontan patients and healthy controls (8). Both studies concluded that short-term exposure and exercise at high altitude was tolerated by Fontan patients which is in line with our results (7, 8). However, myocardial function measured by echocardiography was not addressed in these studies. To our knowledge, only Garcia et al. (23) measured echocardiographic parameters, but results were poor due to inadequate image quality.

### 4.1. Effect of high-altitude hypoxia on systemic ventricular function at rest

On acute exposure to hypoxia, an increase in myocardial contractility, heart rate and cardiac output can normally be observed in healthy individuals due to sympathetic activation (4). In our study, these changes were demonstrated in the healthy control group, but a mild decline in systolic as well as diastolic function was observed at rest in hypoxia compared to baseline values in normoxia in the Fontan group. However, the decrease was mild and the effect of hypoxia on systolic echocardiographic parameters did not reach statistical significance with the except of APSE.

The absent increase in functional echocardiographic parameters at rest in hypoxia in the Fontan group may be due to intrinsic myocardial dysfunction or because of changes in pre- and/or afterload. As demonstrated by peripheral blood pressure measurements, increased afterload is not likely to play a role in short-term altitude exposure as hypoxic vasodilation leads to a mild decline in blood pressure and hence afterload (4). Although we are not able to measure preload by conventional echocardiography, the estimation of ventricular area may be used as a surrogate marker for preload and ventricular filling. Considering the constant area measurements at normoxia and hypoxia, a reduction in preload is unlikely. This is in line with the findings of Staempfli et al. (7). However, both Steampfli et al. (7) and this study applied indirect methods to estimate preload; hence both studies are not able to reliably differentiate whether the lacking rise of myocardial systolic function in Fontan patients is related to intrinsic cardiac dysfunction or preload inadequacy caused by hypoxia. This could potentially be extrapolated by further examinations under hypoxic conditions like magnetic resonance imaging. Generally, echocardiographic assessment of the univentricular circulation remains challenging as many well-known parameters are not applicable in Fontan patients (24, 25). Instead, VTI across the aorta/neo-aorta may be used as a surrogate of stroke volume (26) and VTI × HR as a surrogate of cardiac output (12). Based on this computation of cardiac output and the area measurements, our paper supports the findings of previous studies that high altitude does not lead to a severe reduction of cardiac output in Fontan patients (7).

On acute exposure to hypoxia, ventricular diastolic dysfunction is usually prevented by an augmentation of atrial contraction in healthy individuals (27). In our study, however, the expected increase of late, active diastolic filling (A-wave) at rest in hypoxia was only found in healthy controls and not in the Fontan group. Diastolic dysfunction is commonly observed in Fontan patients, but evaluation remains challenging as standardized and well-studied echocardiographic parameters are still lacking (5, 28).

### 4.2. Effect of high-altitude hypoxia on systemic ventricular function after peak and continuous exercise

According to Claessen et al. (29), inadequate cardiac filling and reductions in systolic contractility may lead to a decline in stroke volume during exercise in normoxia in Fontan patients. As a consequence, exercise-related cardiac output may mainly be augmented by heart rate and not by a combined increase in stroke volume and heart rate as in healthy individuals (29). This may lead to a premature plateau in cardiac output in Fontan patients (29). This may explain why a fall in cardiac output was observed after PE in hypoxia compared to normoxia in Fontan patients, whereas an increase was found in the control group. As discussed by Claessen et al. (29), diastolic active filling may play a role which is in line with our findings. In comparison to healthy controls, augmented atrial contraction in diastolic filling (A-wave) was not observed in the Fontan group after PE. However, a significant reduction in systemic ventricular function after exercise was also not demonstrated as hypothesized, because the changes were mild and Fontan patients clinically tolerated PE in hypoxia. The clinical implications of these findings remain debatable.

To our knowledge, this is the first study to include data after continuous exercise that reflects more realistic physical activity at high altitude such as skiing or hiking. Overall, Fontan patients seem to tolerate CE more than PE at high altitude which is in line with the incapability to increase the necessary cardiac output at higher exercise levels. In this study, an augmentation of VTI as a surrogate of stroke volume was only observed after CE in hypoxia in our Fontan group. Together with a rise in heart rate, this led to an increase in cardiac output after CE but not after PE in hypoxia compared to normoxia. However, further studies including CE are needed.

### 4.3. Limitations

The limitations of this study include its relatively small sample size. It is important to point out that echocardiographic examination was not performed during peak and continuous exercise but was carried out immediately after exercise testing. However, the same protocol was applied to both groups allowing comparison of the results within our study. Furthermore, only Fontan patients with NYHA class I or II were included so that our results cannot be generalized to patients with more severe symptoms and more pronounced ventricular dysfunction. Although it is thought that normobaric hypoxia and hypobaric hypoxia lead to similar responses (30), it should be considered that only normobaric hypoxia was used in this study. The maximal time period at simulated altitude was 120 min in all participants which may not reflect everyday scenarios.

## 5. Conclusion

In hypoxia, a consistent decline in echocardiographic parameters was not observed in patients with univentricular circulation. Short-term exposure to high-altitude hypoxia and exercise was clinically tolerated by this group, but continuous exercise appeared to be better tolerated than peak exercise as measured by echocardiography. However, Fontan patients did not show increased values of myocardial contractility, heart rate and cardiac output at rest and after peak exercise as observed in healthy controls. This is likely to be multifactorial and may include intrinsic cardiac dysfunction as well as preload inadequacy and the lack of augmented atrial contraction. Further studies including stress echocardiography and/or invasive measurement techniques and long-term altitude exposure are needed.

## Data Availability

The original contributions presented in the study are included in the article/**Supplementary material**, further inquiries can be directed to the corresponding author/s.
