# Supplementary material for: Systemic ventricular function in Fontan patients at rest and after exercise at altitude
Source: Front Pediatr. 2023 Jan 6;10:1084468. doi: 10.3389/fped.2022.1084468 (PMC9853047; doi:10.3389/fped.2022.1084468)
Supplement: Supplementary file 1 [file Datasheet1.pdf]

Supplementary Material

1 Supplementary Tables

Table S1. Echocardiographic parameters.

Length diastole (mm)
Length systole (mm)
Length change (%)
Area diastole (cm^2)
Area systole (cm^2)
FAC (%)
VTI (cm)
HR (bpm) at time of VTI
CO (VTI x HR)
APSE (mm)
s' (cm/s)
IVCT (ms)
GLS (%)
E (m/s)
A (m/s)
e' (cm/s)
E/e'
IVRT (ms)
IVRT/RR
IVC CI (%)
MPI

FAC = fractional area change; VTI = velocity time integral over aorta/neo-aorta; HR = heart rate; bpm = beats per minute; CO = cardiac output; APSE = annular plane systolic excursion; IVCT = isovolumic contraction time; GLS = global systolic strain; IVRT = isovolumic relaxation time; IVRT/ RR = isovolumic relaxation time corrected for heart rate; IVC CI = inferior vena cava collapsibility index; MPI = myocardial performance index.

Table S2. Clinical and echocardiographic parameters of Fontan patients and healthy controls at rest and after exercise in normoxia and hypoxia.

		At rest, normoxia	After CE, normoxia	After PE, normoxia	At rest, hypoxia	After CE, hypoxia	After PE, hypoxia
**Oxygen saturation (%)**	Fontan	93.0 (90.5-95.0)	90.5 (87.0-93.0)	91.0 (86.5-92.0)	88.0 (85.0-89.0)	83.0 (81.0-88.0)	84.0 (77.0-86.0)
	Control	99.0 (98.0-100.0)	96.0 (95.0-96.5)	97.0 (96.0-98.5)	93.0 (92.0-95.5)	89.0 (88.0-90.0)	90.0 (87.0-94.0)
**Systolic BP (mmHg)**	Fontan	113.6 ± 10.3	133.4 ± 13.1	154.9 ± 23.0	114.3 ± 14.4	126.5 ± 12.9	150.0 ± 27.4
	Control	124.2 ± 12.7	158.2 ± 28.0	171.5 ± 31.8	122.6 ± 12.5	154.9 ± 25.0	176.0 ± 28.4
**Diastolic BP (mmHg)**	Fontan	74.8 ± 10.6	64.1 ± 11.1	72.8 ± 11.5	72.3 ± 12.7	73.0 ± 13.5	71.2 ± 11.8
	Control	76.3 ± 11.3	61.9 ± 12.5	72.4 ± 17.5	73.3 ± 9.4	63.0 ± 13.6	71.5 ± 18.5
**Length diastole (mm)**	Fontan	65.9 ± 11.1	64.9 ± 9.7	64.5 ± 11.3	67.9 ± 9.8	67.5 ± 9.8	67.8 ± 9.6
	Control	80.4 ± 8.1	78.5 ± 8.0	77.1 ± 6.3	78.9 ± 7.8	78.1 ± 7.5	80.5 ± 7.5
**Length systole (mm)**	Fontan	56.0 ± 11.1	55.3 ± 11.0	55.1 ± 11.2	59.2 ± 10.7	56.2 ± 9.9	56.7 ± 10.4
	Control	63.2 ± 8.2	61.9 ± 7.9	59.7 ± 7.1	62.4 ± 8.3	62.2 ± 6.2	63.5 ± 8.5
**Length change (%)**	Fontan	15.3 (10.8-21.3)	13.0 (12.0-17.6)	14.7 (9.7-20.5)	11.8 (9.8-15.7)	16.1 (13.7-20.1)	15.0 (12.6-17.9)
	Control	22.1 (18.6-24.2)	21.5 (19.0-23.6)	24.7 (19.5-27.2)	21.7 (18.7-24.3)	21.0 (18.5-22.8)	21.5 (16.9-23.8)
**Area diastole (cm²)**	Fontan	30.1 (27.7-37.4)	30.6 (27.9-34.6)	30.7 (26.5-35.5)	31.7 (28.0-34.9)	29.5 (25.0-33.8)	29.9 (25.6-37.8)
	Control	32.1 (29.3-38.4)	31.8 (28.9-36.0)	30.4 (27.8-35.7)	31.9 (27.5-39.6)	32.4 (29.5-37.0)	33.1 (28.6-38.4)
**Area systole (cm²)**	Fontan	17.6 (11.7-23.6)	17.3 (13.8-22.5)	18.2 (13.5-22.6)	18.8 (13.4-24.2)	17.6 (13.3-19.4)	17.7 (13.2-22.5)
	Control	18.9 (15.6-23.6)	18.5 (17.2-20.1)	18.6 (14.5-19.9)	16.9 (15.2-22.6)	18.5 (15.4-21.2)	18.9 (15.8-21.9)
Parameters of systolic function							
**FAC (%)**	Fontan	40.5 (36.7-46.1)	40.5 (37.2-47.4)	39.5 (34.2-44.5)	39.2 (34.8-45.8)	41.5 (37.8-49.7)	40.6 (36.0-44.9)
	Control	41.9 (37.6-44.7)	41.7 (38.2-45.6)	42.9 (38.8-47.8)	42.9 (34.4-48.3)	42.5 (37.7-47.4)	43.7 (38.3-46.1)
**VTI (cm)**	Fontan	19.43 (15.00-24.30)	18.18 (16.87-18.93)	19.00 (16.67-23.47)	19.63 (15.83-24.30)	18.07 (15.37-20.53)	19.03 (15.50-21.70)
	Control	23.58 (20.73-26.07)	25.07 (23.23-27.30)	21.10 (19.93-22.77)	24.75 (22.20-25.83)	24.83 (21.13-28.77)	24.27 (21.23-25.37)
**HR (bpm) at time of VTI**	Fontan	67 ± 10	79 ± 10	78 ± 14	65 ± 14	85 ± 16	81 ± 16
	Control	69 ± 12	82 ± 11	96 ± 12	71 ± 9	85 ± 12	98 ± 11
**CO (VTI x HR)**	Fontan	1460 (1095-1677)	1411 (1339-1472)	1539 (1274-1816)	1362 (1069-1495)	1520 (1347-1828)	1534 (1310-1659)
	Control	1453 (1300-1827)	2080 (1729-2253)	2023 (1696-2254)	1622 (1487-1762)	2056 (1733-2610)	2268 (1986-2540)
**APSE (mm)**	Fontan	11.96 (10.52-14.48)	14.83 (12.18-15.50)	12.79 (11.45-14.77)	11.03 (9.30-14.12)	11.90 (9.30-14.00)	10.68 (8.51-13.73)
	Control	16.28 (15.69-17.96)	16.28 (14.62-18.54)	15.79 (14.45-16.94)	16.91 (14.55-18.53)	17.52 (15.48-19.06)	15.38 (14.15-16.24)
**s' (cm/s)**	Fontan	6.93 (6.05-7.64)	7.70 (7.07-8.74)	6.90 (6.20-8.52)	7.18 (6.01-7.90)	7.79 (7.02-8.30)	8.07 (7.20-8.49)
	Control	10.17 (9.17-11.95)	11.66 (11.05-12.63)	12.20 (11.05-13.85)	10.90 (9.56-12.68)	12.39 (10.33-14.60)	13.31 (11.08-15.20)
**IVCT (ms)**	Fontan	62.0 (56.5-88.0)	61.5 (60.5-67.0)	64.0 (58.0-82.0)	67.0 (59.0-88.0)	71.5 (67.8-79.0)	71.3 (58.5-74.8)
	Control	68.0 (50.5-77.5)	59.0 (52.0-65.8)	64.0 (58.0-73.0)	64.0 (58.0-71.5)	57.0 (53.5-67.0)	53.5 (52.0-59.5)
**GLS (%)**	Fontan	-22.81 ± 4.78	-20.71 ± 3.05	-22.12 ± 4.03	-20.49 ± 4.39	-20.77 ± 5.40	-20.91 ± 5.27
	Control	-22.61 ± 3.63	-22.91 ± 3.76	-23.53 ± 3.97	-22.17 ± 3.09	-23.03 ± 2.97	-21.94 ± 2.59
Parameters of diastolic function							
**E (m/s)**	Fontan	0.70 (0.61-0.81)	0.75 (0.72-0.85)	0.80 (0.74-0.90)	0.74 (0.66-0.79)	0.75 (0.64-0.93)	0.81 (0.63-0.92)
	Control	0.98 (0.76-1.05)	0.91 (0.75-1.01)	0.90 (0.81-1.00)	0.89 (0.80-1.07)	0.95 (0.73-1.01)	0.89 (0.79-0.94)
**A (m/s)**	Fontan	0.51 (0.36-0.57)	0.50 (0.43-0.56)	0.50 (0.37-0.65)	0.49 (0.37-0.58)	0.61 (0.39-0.73)	0.53 (0.41-0.63)
	Control	0.40 (0.35-0.55)	0.65 (0.46-0.74)	0.68 (0.56-0.88)	0.51 (0.41-0.62)	0.55 (0.49-0.64)	0.81 (0.64-0.97)
**e' (cm/s)**	Fontan	11.41 ± 3.10	12.90 ± 3.88	13.18 ± 4.33	10.95 ± 2.51	13.23 ± 3.84	13.19 ± 3.03
	Control	17.54 ± 2.24	17.34 ± 2.95	16.96 ± 3.10	17.39 ± 3.09	16.91 ± 2.90	16.75 ± 3.01
**E/e'**	Fontan	6.31 (4.88-9.01)	6.34 (5.25-8.21)	7.28 (5.62-9.42)	6.78 (5.12-8.45)	5.26 (5.19-8.64)	5.58 (5.38-6.89)
	Control	5.54 (4.90-5.73)	4.98 (4.88-5.67)	5.37 (5.09-5.68)	5.54 (4.31-6.76)	5.17 (4.91-5.96)	5.02 (4.74-5.76)
**IVRT (ms)**	Fontan	65.5 (61.0-80.5)	62.5 (59.5-81.5)	70.0 (55.0-86.5)	65.5 (56.5-74.5)	67.5 (62.5-76.0)	68.0 (62.5-74.5)
	Control	58.0 (53.5-71.0)	52.5 (46.8-56.5)	52.0 (43.0-59.0)	59.0 (49.0-67.0)	55.0 (51.5-58.5)	53.5 (46.7-59.5)
**IVRT/ RR**	Fontan	0.08 ± 0.02	0.09 ± 0.03	0.09 ± 0.03	0.08 ± 0.02	0.10 ± 0.02	0.09 ± 0.02
	Control	0.07 ± 0.02	0.08 ± 0.02	0.08 ± 0.02	0.07 ± 0.02	0.08 ± 0.01	0.08 ± 0.01
**IVC CI (%)**	Fontan	21.6 (14.4-33.8)	22.1 (15.3-25.5)	16.2 (13.1-22.3)	27.7 (17.4-38.7)	18.3 (13.0-24.2)	21.3 (13.7-32.4)
	Control	55.4 (37.2-64.1)	61.2 (50.7-69.9)	58.0 (41.0-69.0)	49.6 (41.6-61.4)	64.9 (45.1-75.7)	58.7 (44.3-71.3)
Global ventricular function							
**MPI**	Fontan	0.44 (0.38-0.52)	0.45 (0.42-0.51)	0.51 (0.43-0.61)	0.49 (0.40-0.54)	0.55 (0.47-0.63)	0.48 (0.43-0.57)
	Control	0.41 (0.36-0.47)	0.40 (0.36-0.45)	0.42 (0.37-0.53)	0.42 (0.38-0.46)	0.41 (0.37-0.42)	0.40 (0.37-0.42)

Data are expressed as mean ± SD or median (IQR).
CE = continuous exercise; PE = peak exercise; BP = blood pressure; FAC = fractional area change; VTI = velocity time integral over aorta/neo-aorta; HR = heart rate; bpm = beats per minute; CO = cardiac output; APSE = annular plane systolic excursion; IVCT = isovolumic contraction time; GLS = global systolic strain; IVRT = isovolumic relaxation time; IVRT/ RR = isovolumic relaxation time corrected for heart rate; IVC CI = inferior vena cava collapsibility index; MPI = myocardial performance index.

Table S3. Intraobserver and interobserver variability.

	Intraobserver variability		Interobserver variability	
Length diastole (mm)	0.987	(0.967 to 0.995)	0.918	(0.407 to 0.978)
Length systole (mm)	0.988	(0.962 to 0.996)	0.884	(0.489 to 0.963)
Area diastole (cm^2)	0.998	(0.995 to 0.999)	0.972	(0.917 to 0.990)
Area systole (cm^2)	0.994	(0.985 to 0.998)	0.969	(0.918 to 0.988)
VTI (cm)	0.996	(0.984 to 0.999)	0.978	(0.938 to 0.992)
APSE (mm)	0.979	(0.948 to 0.992)	0.960	(0.889 to 0.986)
s’ (cm/s)	0.999	(0.996 to 0.999)	0.994	(0.963 to 0.998)
GLS (%)	0.837	(0.583 to 0.937)	0.867	(0.654 to 0.950)
E (m/s)	0.984	(0.951 to 0.994)	0.991	(0.977 to 0.996)
A (m/s)	0.998	(0.987 to 0.999)	0.997	(0.993 to 0.999)
e’ (cm/s)	0.998	(0.995 to 0.999)	0.985	(0.958 to 0.995)
E/e’	0.989	(0.964 to 0.996)	0.989	(0.964 to 0.996)

Data are presented as intraclass correlation with 95% confidence interval.

2 Supplementary Figures

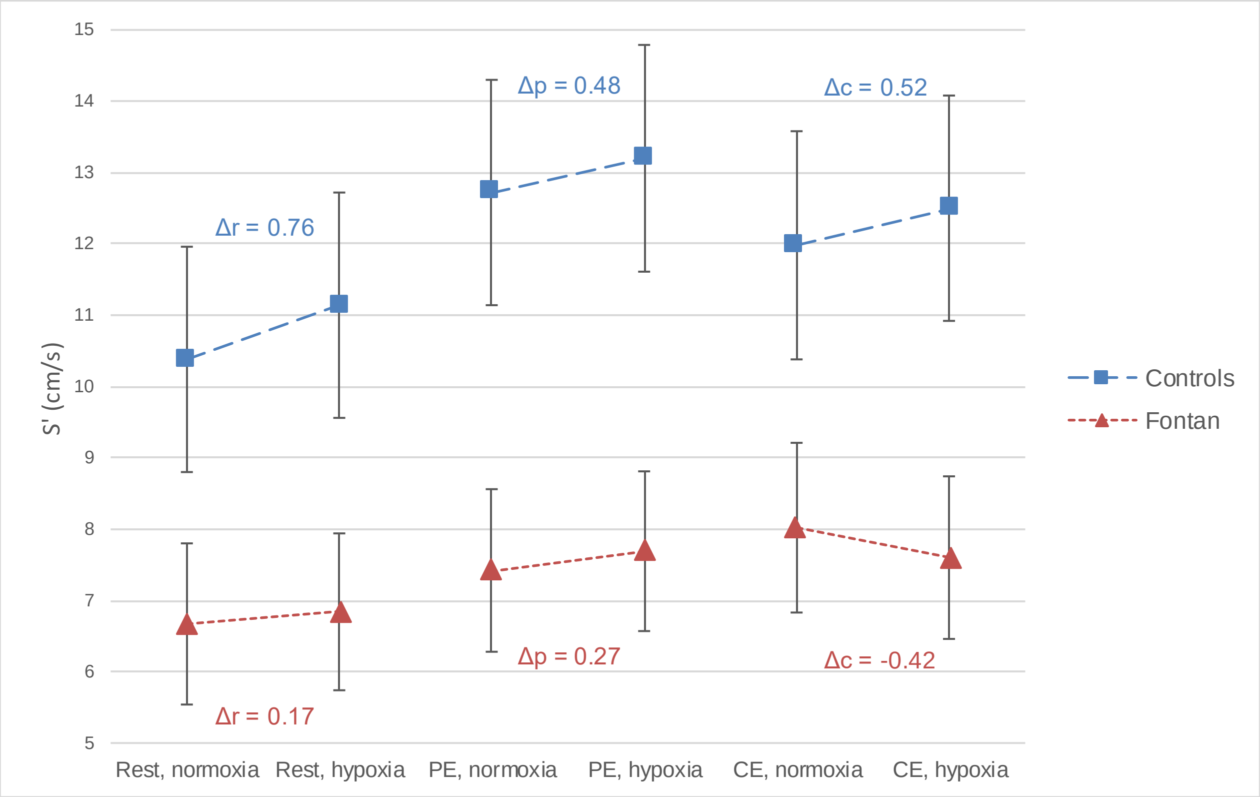

Supplementary Figure 1. Effect of exercise and hypoxia on s' in Fontan patients and controls analyzed by linear mixed-effects model (LMM). A statistically significant effect of exercise on s' was found in healthy controls ($p<0.001$).
Δ= (mean predicted s' in hypoxia – mean predicted s' in normoxia) in cm/s; calculated for each exercise level and group.

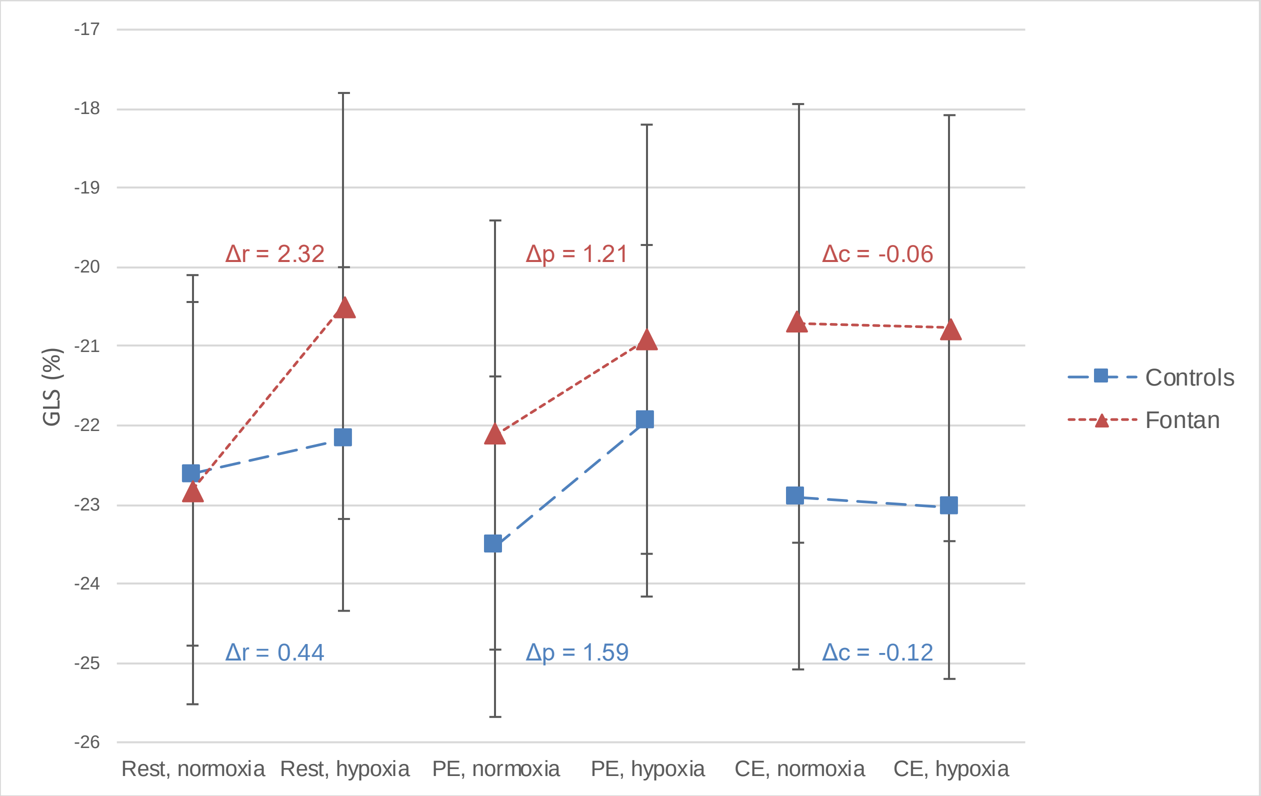

Supplementary Figure 2. Effect of exercise and hypoxia on global longitudinal strain (GLS) in Fontan patients and controls analyzed by LMM. No statistically significant effects of exercise or hypoxia on GLS were found in either group.
Δ= (mean predicted GLS in hypoxia – mean predicted GLS in normoxia) in %; calculated for each exercise level and group.

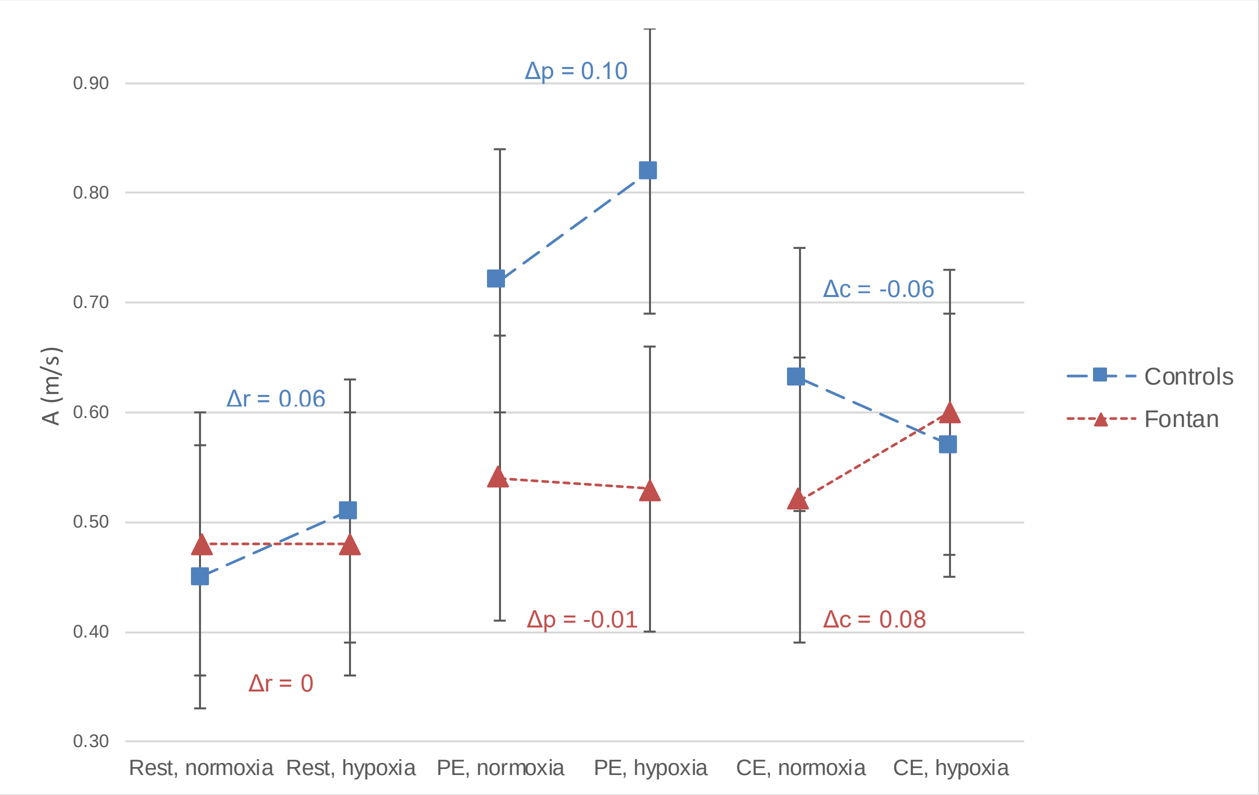

Supplementary Figure 3. Effect of exercise and hypoxia on A-wave velocity in Fontan patients and controls analyzed by LMM. A statistically significant effect of exercise on A-wave was found in healthy controls ($p<0.001$).
Δ= (mean predicted A in hypoxia – mean predicted A in normoxia) in m/s; calculated for each exercise level and group.

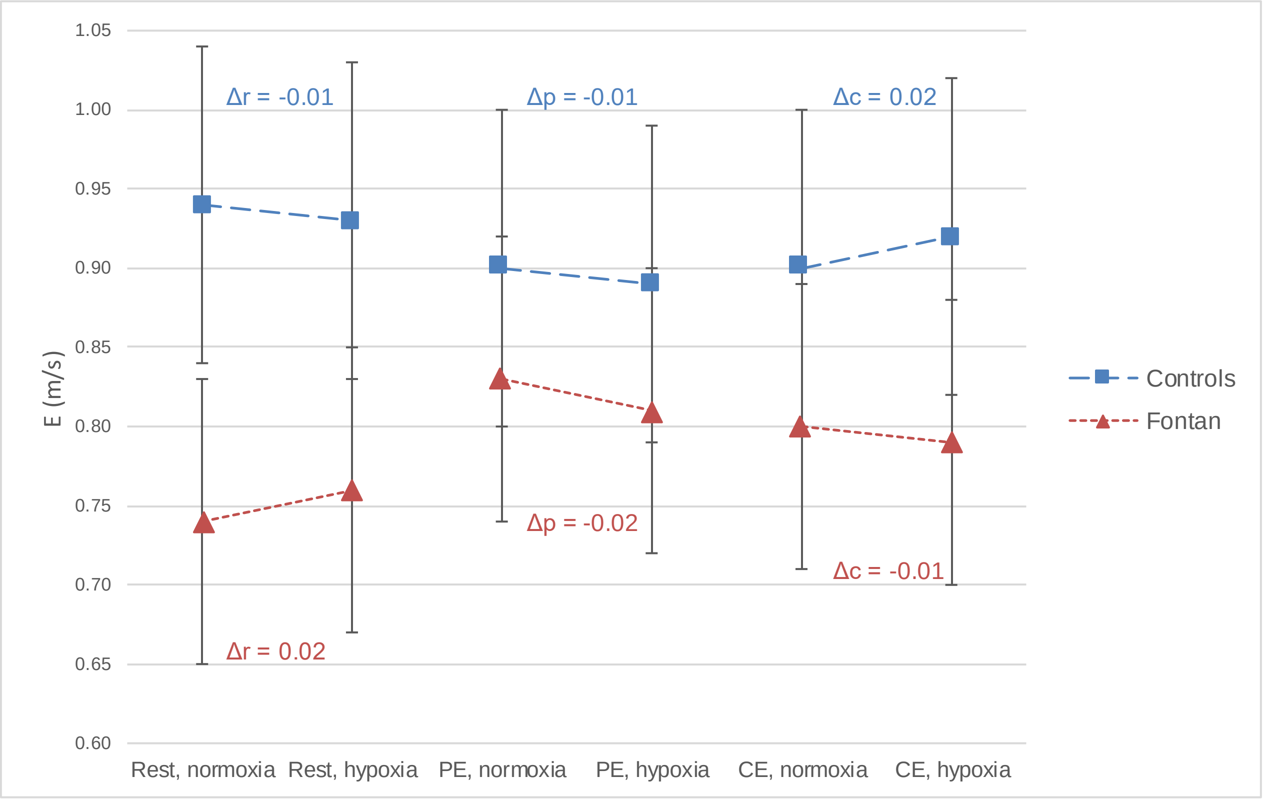

Supplementary Figure 4. Effect of exercise and hypoxia on E-wave in Fontan patients and controls analyzed by LMM. No statistically significant effects of exercise or hypoxia on E-wave were found in either group.
Δ= (mean predicted E in hypoxia – mean predicted E in normoxia) in m/s; calculated for each exercise level and group.

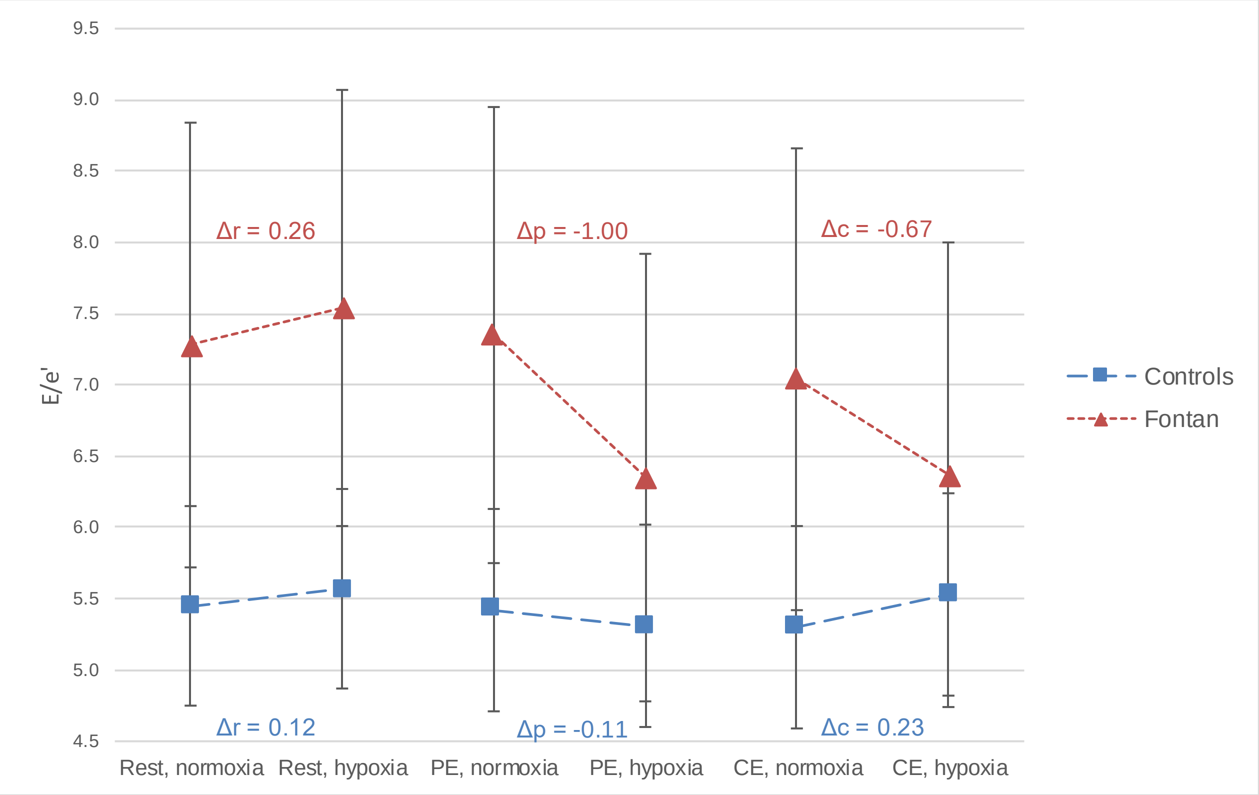

Supplementary Figure 5. Effect of exercise and hypoxia on E/e' ratio in Fontan patients and controls analyzed by LMM. No statistically significant effects of exercise or hypoxia on E/e' ratio were found in either group.
Δ= (mean predicted E/e' in hypoxia – mean predicted E/e' in normoxia); calculated for each exercise level and group.
